# IMPaCT Back study protocol. Implementation of subgrouping for targeted treatment systems for low back pain patients in primary care: a prospective population-based sequential comparison

**DOI:** 10.1186/1471-2474-11-186

**Published:** 2010-08-20

**Authors:** Nadine E Foster, Ricky Mullis, Julie Young, Carol Doyle, Martyn Lewis, David Whitehurst, Elaine M Hay

**Affiliations:** 1 Arthritis Research UK Primary Care Centre, Primary Care Sciences, Keele University, Staffordshire, ST5 5BG, UK; 2 General Practice & Primary Care Research Unit, Department of Public Health & Primary Care, University of Cambridge, UK; 3 Central and Eastern Cheshire Primary Care Trust, Universal House, Middlewich, Cheshire, UK; 4 Health Economics Unit, School of Health and Population Sciences, University of Birmingham, Edgbaston, Birmingham, UK

## Abstract

**Background:**

Prognostic assessment tools to identify subgroups of patients at risk of persistent low back pain who may benefit from targeted treatments have been developed and validated in primary care. The IMPaCT Back study is investigating the effects of introducing and supporting a subgrouping for targeted treatment system in primary care.

**Methods/Design:**

A prospective, population-based, quality improvement study in one Primary Care Trust in England with a before and after design. Phases 1 and 3 collect data on current practice, attitudes and behaviour of health care practitioners, patients' outcomes and health care costs. Phase 2 introduces and supports the subgrouping for targeted treatment system, via a multi-component, quality improvement intervention that includes educational courses and outreach visits led by opinion leaders, audit/feedback, mentoring and organisational support to embed the subgrouping tools within IT and clinical management systems.

We aim to recruit 1000 low back pain patients aged 18 years and over consulting 7 GP practices within one Primary Care Trust in England, UK. The study includes GPs in participating practices and physiotherapists in associated services. The primary objective is to determine the effect of the subgrouping for targeted treatment system on back pain related disability and catastrophising at 2 and 6 months, comparing data from phase 1 with phase 3. Key secondary objectives are to determine the impact on:

a) GPs' and physiotherapists' attitudes and behaviour regarding low back pain;

b) The process of care that patients receive;

c) The cost-effectiveness and sustainability of the new clinical system.

**Discussion:**

This paper details the rationale, design, methods, planned analysis and operational aspects of the IMPaCT Back study. We aim to determine whether the new subgrouping for targeted treatment system is implemented and sustained in primary care, and evaluate its impact on clinical decision-making, patient outcomes and costs.

**Study registration:**

International Standard Randomised Controlled Trial Number Register ISRCTN55174281

## Background

Low back pain (LBP) affects over one third of adults at any one time, and each year approximately 3.5 million people in the UK develop back pain [1]. It is the most common reason for middle-aged people to visit their general practitioner (GP), with approximately 6-9% of adults consulting for this condition each year [2]. Although many back pain patients stop consulting their GP within three months, 60-80% of people still report pain or disability a year later, and up to 40% of those who have taken time off work will have future episodes of work absence [3,4]. The societal cost of work absence attributable to back pain, together with back pain related health care utilisation, constitutes a considerable economic burden. Total back pain related costs in the UK are estimated to form 1-2% of gross national product, [5] with National Health Service (NHS) related costs alone in the region of £251 million per annum [1].

Most patients with back pain are treated in primary care, where an estimated 85% will have 'non-specific' LBP, for which diagnostic labelling is discouraged, and treatment is guided by symptoms and the experience and preferences of individual health care practitioners. In the UK, referral to physiotherapy is a popular management option for GPs, with LBP accounting for more than half of physiotherapists' workload [6]. Whilst the focus of primary care treatment is on minimising pain and disability, previous studies have shown variation in clinical practice [7] and highlighted the gap between current practice and best practice recommendations [6,8-12]. Previous studies have also underlined the challenge practitioners face in applying best practice at an individual patient level, their concerns about the need for specialist advice for those with chronic LBP [13] and their capacity to identify and address psychological obstacles to recovery [14].

Patients with poorer physical function and those with psychological obstacles to recovery such as psychological distress, negative feelings about their back pain and increased fear of activity, are more disabled by their pain and are more likely to have a poor outcome [15]. Despite the plethora of clinical guidelines for the management of back pain and the call to use a biopsychosocial framework [15], a key challenge is the early identification of patients at risk of chronicity and subsequently preventing such chronicity [16]. Addressing these factors in primary care at an early stage before they become entrenched and more difficult to treat could lead to better long-term outcomes.

Improved patient outcomes have been demonstrated in some studies where subgrouping has been used to guide treatment [17,18]. Prognostic assessment tools, in primary care, to identify subgroups of patients at risk of persistent LBP and who may benefit from interventions that target key physical and/or psychological obstacles to recovery have been developed and validated. The STarT Back tool [19] is specifically designed for primary care settings and is a subgrouping tool that classifies patients into three categories for targeted treatment, based on the presence of modifiable risk factors for chronic or recurrent LBP. Three targeted treatments for patients and training programmes for clinicians have been developed. A randomised controlled trial (the STarT Back trial) has tested whether subgrouping for targeted treatment is better than best current care (provided by physiotherapists) of non-targeted treatment [20] addressing the call within recent national guidelines for further investigation of 'matching' or subgrouping LBP patients to different treatments [15].

The IMPaCT Back study (IMplementation study to improve Patient Care through Targeted treatment for Back pain) is a quality improvement study designed to introduce and support a subgrouping for targeted treatment system within primary care practice, and to study the effects on patients, practitioners and health care resource use. Many terms are used to describe the process of quality improvement in clinical practice, such as implementation, knowledge diffusion, transfer and exchange, and innovation diffusion. Implementation research is defined as '*the scientific study of methods to promote the systematic uptake of research findings and other evidence-based practices into routine practice, to improve the quality of health care. It includes the study of influences on healthcare professional and organisation behaviour' *[21]. In this paper, we use the term quality improvement as in the SQUIRE (Standards for Quality Improvement Reporting Excellence) guidelines [22] in recognition that this is a process that needs a systematic and planned approach but that it is also essentially an applied science, driven by experiential learning so that interventions can be modified in response to feedback.

Unlike targeting the general public through mass media campaigns about LBP [23-25] we are targeting health care practitioners and the health care system within which they work. Several previous studies have attempted to change health care practitioner behaviour for LBP [11,26-31] with mixed results. None have investigated subgrouping for targeted treatment systems based on risk identification or provided comprehensive data on the effect of the quality improvement on practitioners' attitudes and behaviours, clinical processes of care, patients' clinical outcomes and cost-effectiveness.

### Study Aims

Working with one Primary Care Trust (PCT) in England, we will introduce a subgrouping for targeted treatment care system for the assessment and management of LBP patients in primary care and evaluate the effects on health care practitioner attitudes and behaviour, care processes, patients' clinical outcomes through to six months following their primary care consultation and cost-effectiveness. Specific objectives are to:

(i) engage GPs, physiotherapists and PCT managers to deliver the new subgrouping for targeted treatment model of care;

(ii) change GPs' and physiotherapists' LBP-related attitudes and clinical behaviours using the subgrouping for targeted treatment approach;

(iii) improve patients' clinical outcomes at 2 and 6 months follow-up;

(iv) estimate the cost-effectiveness of the subgrouping approach, addressing NHS and societal interests;

(v) provide evidence for the sustainability of this care system.

This publication details the rationale, design, the subgrouping for targeted treatment system, study methods, operational aspects and planned analysis of the IMPaCT Back study.

## Methods

### Study Design

A prospective, population-based, quality improvement study of before and after design, comprising three phases:

Phase 1: assessment of GPs' and physiotherapists' attitudes and behaviours regarding LBP, observation of usual clinical practice and clinical outcomes of patients recruited in a 6 month baseline period;

Phase 2: a multi-component, quality improvement intervention comprising educational courses and outreach visits led by opinion leaders, regular audit/feedback and mentoring support for participating health care practitioners in addition to installation of computerised and paper-based systems to support the subgrouping for targeted treatment care system in practice;

Phase 3: assessment of GPs' and physiotherapists' attitudes and behaviours regarding LBP, observation of clinical practice and clinical outcomes of patients, recruited in a 12 month period following roll-out of the new care system.

**Figure 1:**
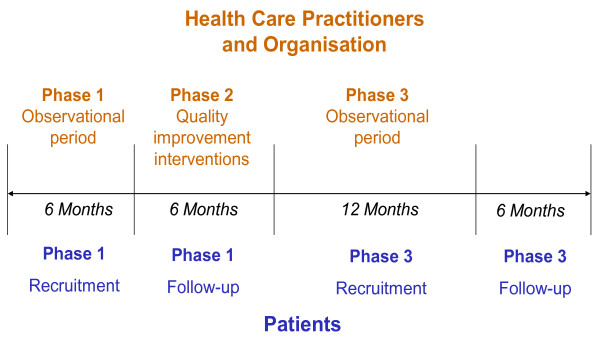
Summary of IMPaCT Back study design

The study design is summarised in Figure 1.

We considered using a clinical trial design with cluster randomisation at the level of GP practice. However, the physiotherapy service is PCT based rather than GP practice based, which would have been likely to lead to contamination of clusters.

### Setting

Participants will be recruited from up to 7 GP practices and their associated physiotherapy services within one NHS PCT in the county of Cheshire, England, UK. An NHS PCT is a type of NHS Trust, part of the NHS in England, that provides primary and community services or commissions them from other providers, and is involved in commissioning secondary care [32]. There are approximately 152 PCTs in England.

### Ethical review

Favourable ethical opinion was obtained from the Cheshire Local NHS Research Ethics Committee (Study number: 07/H1017/143).

### Independent monitoring

A steering committee with an independent chair and lay representation is monitoring the study's progress.

### Participants

Health care practitioners (GPs and physiotherapists) and patients who consult them with LBP are considered participants in this study.

### Health care practitioners' eligibility criteria

All practising GPs at each participating practice and physiotherapists within the associated physiotherapy services receiving LBP referrals from the participating GP practices are eligible for the study. We will select which GP practices within the PCT to invite in order to ensure a breadth of practice settings (urban/semi-rural/rural) and size (small/medium/large). General practices and physiotherapy services are invited to participate following presentations by the study team at meetings of their practice and physiotherapy staff. Strategies to facilitate participation of general practices and physiotherapy services in the study include continuing professional development activities, receiving guidance and feedback on their practice, and the provision of up to date knowledge, skills and tools to support their management of LBP patients.

### Patients' eligibility criteria

Male and female adults aged 18 years or over, consulting one of the participating GP practices with non-specific LBP (with or without leg pain). Participants must understand English to a level where they can read the study information leaflet and study questionnaires. Exclusions are those with "red flags" (indicative of possible serious spinal pathology such as cauda equina, inflammatory arthritis, malignancy, infection, fracture); pregnancy-related LBP; conditions which might exclude the patient from physiotherapy treatment (e.g. serious co-morbidity, recent major surgery), patients already receiving physiotherapy treatment for this episode of LBP.

The procedures for recruitment, assessment and treatment are summarised below for each phase of the IMPaCT Back study.

### Phase 1: Observation of usual clinical practice and patients' outcomes

We will collect data on health care practitioners' back pain-related attitudes and behaviours, patients' clinical outcomes and health care resource use before the introduction of the new subgrouping for targeted treatment system. Data collection in phase 1 will consist of:

#### a) Health care practitioners' attitudes and behaviour and care processes

Attitudes and reported behaviour: We will conduct a questionnaire survey of participating GPs and physiotherapists to describe their attitudes and beliefs about back pain and its management, and their reported clinical behaviour relating to two clinical case vignettes developed from real patients with LBP. The questionnaire and case vignettes have been adapted from a previous national survey of GPs and physiotherapists [7]. The self-report questionnaire will include key demographic and clinical experience questions, a measure of beliefs and attitudes towards LBP, the Pain Attitudes and Beliefs Scale (PABS) [33,34], and a measure of self-confidence in managing LBP patients [35]. Full details of the contents of the questionnaire are provided in Table 1.

**Table 1:** Contents of Health Care Practitioner Questionnaire

Domain	Outcome Measures	Study Phase
**Clinical experience**	Length of time qualified	1
	Post-graduate training	1,2,3
**Self-confidence**	Self-confidence in managing back pain patients [35]	1,2,3
**Beliefs and attitudes**	Pain Attitudes and Beliefs Scale (PABS) [33,34]	1,2,3
**Reported clinical behaviour**	Clinical case vignettes of patients with non-specific low back pain with items relating to investigations, referrals, advice and medication	1,2,3
	Changes in management of low back pain following best practice updates/training programme	2,3
**Education**	Future learning needs	2,3

Care processes and actual clinical behaviour: We will collect data on health care practitioners' actual clinical behaviour from anonymised GP medical record reviews and physiotherapy case report forms, for patients who consent to the study team accessing their medical records. We aim to collect data on primary care consultations, ordering of diagnostic tests (radiographs, MRI and CT scans), prescribed medications, referrals to other professionals or services and issuing of sickness certificates. Additional data from physiotherapy case report forms will include the physiotherapist's view of the patient's key problems and targets for treatment, the treatment approaches used, the advice given and the number and length of treatment sessions.

#### b) Patients' clinical outcomes

A consecutive sample of patients consulting with LBP at each participating practice will be recruited in phase 1 for a period of up to 6 months. As each patient consults with LBP at one of the participating GP practices and is assessed, if their GP enters a previously validated back pain Read Code [2] to indicate the reason for consultation as non-specific LBP, a "pop-up" computer prompt will remind them that the patient is eligible to be invited to participate in the IMPaCT Back study. The GP will inform the patient about the study and ask them if they are willing to receive further information by post. On the same computer pop-up screen the GP will record whether the patient is willing to receive further information about the study, in addition to recording their own clinical impression of the patient's risk of poor outcome (low, medium or high risk of poor outcome). The GP will be able to "opt out" of the computer screen pop-up if they consider the patient not eligible for the study. In phase 1, all patients will receive usual primary care, with referral to other services (including physiotherapy) as usual. There will be no subgrouping for targeted treatment in phase 1, or any specific training or additional resources for assessing and managing LBP patients.

**Figure 2:**
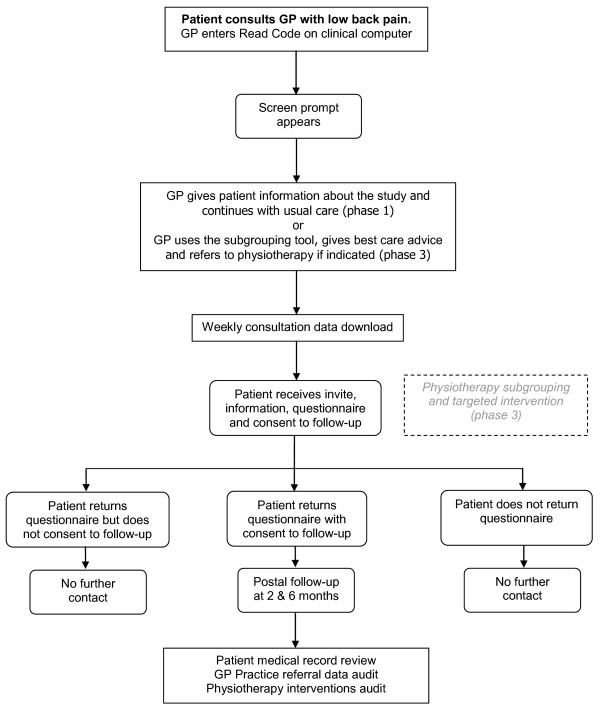
Flow chart of IMPaCT Back study patient recruitment and follow-up

Names and addresses of eligible patients will be extracted weekly from the GP practices' electronic databases. These patients will be sent a letter inviting them to participate in the IMPaCT Back study, an information leaflet and a baseline questionnaire, with a pre-paid return envelope. Participants will be asked to record whether they are willing to be contacted again with follow-up questionnaires at 2 and 6 months, and whether they give permission for their medical records to be reviewed. Figure 2 provides a summary of the patient recruitment and follow-up procedures. The patient questionnaire will collect data across the key domains recommended for LBP studies i.e. back specific function, generic health status, pain, psychological distress, work disability and patient satisfaction [36]. Patient outcome measures are summarised in Table 2. As the main focus is the secondary prevention of long-term disability due to LBP, the primary clinical outcomes are change in the key physical and psychological risk factors for chronicity: back-related disability (Roland-Morris Disability Questionnaire (RMDQ) [37]) and catastrophising (sub-scale from the Coping Strategies Questionnaire [38]) at 6 months.

**Table 2:** Outcome Measures for Patients

	Domain	Outcome Measures	Time Point (months)
**Primary**	**Back Pain Disability**	Roland and Morris Disability Questionnaire [37]	0, 2, 6
	**Catastrophising**	Catastrophising (from Coping Strategies Questionnaire) [38]	0, 2, 6
**Secondary**	**Kinesiophobia**	Tampa Scale Kinesiophobia [53]	0, 6
	**Bothersomeness**	Bothersomeness [2]	0, 2, 6
	**Anxiety/depression**	Hospital Anxiety and Depression Scale [54]	0, 6
	**Self-efficacy**	Pain Self-Efficacy Questionnaire [55]	0, 6
	**Illness perceptions**	Musculoskeletal Illness Perceptions Questionnaire [56]	0, 2, 6
	**Health-related quality of life**	Short Form 12 (version 2) [57]	0, 2, 6
	**Individualised**	Goal Scaling [58]	0, 2, 6
	**Global change**	Compared to symptoms at baseline	2,6
	**Back Pain**	Pain intensity: 0-10 numerical rating scales of least and average pain in last 2 weeks and current pain	0, 2, 6
		Duration [59]	0
	**Pain elsewhere**	Other body region pain(s)	0
	**Preference-based health utility**	EQ-5D [60]	0, 2, 6
	**Employment details**	Current employment status	0,6
		Current/most recent job title	0,6
		Work loss due back problem	0,6
		Work performance	0,6
	**Patient satisfaction**	With information received	2,6
		With care received	2,6
		Rating of overall results of care	2,6
	**Health care resource use**	Primary care consultations	6
		Secondary care attendances (NHS and private)	6
		Additional health care practitioners (NHS and private)	6
		**Prescription and over-the-counter medicines and treatments**	**6**

#### c) Outcomes for the estimation of cost-effectiveness

Recent UK guidelines for the appraisal of cost-effectiveness require changes in health-related quality of life to be based on public preferences using a choice-based method [39]. The EuroQol EQ-5D, a preference-based measure of health status, meets these requirements [40]. The EQ-5D will be collected via the patients' self-report questionnaires at baseline, 2 months and 6 months. Health care resource use data will be collected through a combination of medical record reviews and the 6 month self-report questionnaire. Data collection will focus on key cost drivers including hospital attendances (inpatient stays, outpatient appointments and any other hospital visits to health care practitioners) within the NHS and private practice, consultations with NHS primary health care providers (e.g. general practitioner, practice nurse), prescribed medications, and over-the-counter treatments. Unit costs assigned to these resources will be obtained from published sources reflecting UK national averages. Across all cost components, data will be collected in a disaggregated format that will enable us to estimate, separately, back pain-related costs and costs for 'other' health problems [41]. To address the societal costs of LBP, beyond those directly applicable to health care provision, data regarding employment status and work absence will also be collected.

For those participants that have given permission for their medical records to be reviewed, data will be collected in order to gain further insight into the referral patterns and behaviours of GPs and the self-reported secondary care episodes reported in the 6 month questionnaire. The medical record data will also allow us to assess the validity of responses to some of the resource use questions within the 6 month questionnaire.

### Phase 2: The subgrouping for targeted treatment system and the quality improvement interventions

In phase 2 we will introduce new care systems within the GP practices and physiotherapy services, using a multi-component quality improvement intervention, to train and support participating health care practitioners to use a new subgrouping for targeted treatment system for LBP patients. Details of the subgrouping tools (19) and the subgrouping for targeted treatment system (20) are already available. Briefly, the key features are:

i) subgrouping tools (simple, electronic and paper-based versions) that classify patients as at low, medium or high risk of poor outcome, to help guide clinical decision-making about treatment and onward referral [19];

ii) subgrouping patients on the basis of potentially modifiable risk factors for chronicity;

iii) targeted treatments in primary care for those classified at low, medium and high risk of chronicity. Within the GP consultation, the targeted treatments include a minimal intervention delivered by GPs (for those patients at low risk of poor outcome), or referral to primary care physiotherapists (for those at medium and high risk of poor outcome). Within the physiotherapy consultation, the targeted treatments include a minimal intervention delivered by physiotherapists (for those patients at low risk of poor outcome at the time of physiotherapy consultation), the use of physical approaches for pain relief and physical function (for those at medium risk), and additional cognitive-behavioural approaches to tackle psychological obstacles to recovery (for those at high risk),

iv) multi-component, quality improvement interventions including educational courses and outreach visits led by opinion leaders, regular audit/feedback and mentoring support for GPs and physiotherapists in addition to organisational support to embed the subgrouping tools within existing IT and clinical management systems.

### The sub-grouping tools and targeted treatment approaches

Two versions of a previously developed and validated subgrouping tool for use in primary care [19] will be used. The tools include key physical and psychological predictors of outcome in LBP. A 6-item computer-based subgrouping tool will be embedded in the EMIS GP computer system, for use in real-time GP/patient consultations. Following completion, patients are categorised as either at low (a score of 2 or less) or high (a score of 3 or more) risk of poor outcome and a recommendation is made about whether or not a referral to physiotherapy is likely to be most appropriate. Patients classified as at low risk of poor outcome are recommended to receive best primary care advice and management by the GP, to include a screen for red flags, reassurance about their good prognosis, the benign nature of their pain, and simple messages and advice about pain relief, appropriate physical activity levels, return to normal activity (including work), avoiding bed rest, appropriate use of pain relieving modalities and the role of further investigations. To reinforce these key messages, a brief information sheet will be given. No onward referral to other services is recommended although patients will be advised to re-consult if symptoms persist. Patients at high risk of poor outcome are recommended to be referred to physiotherapy. The GP and patient are then able to use this recommendation in their decision-making.

A 9-item paper-based version of the subgrouping tool will be used by physiotherapists to categorise patients as at low (a score of 3 or less), medium (score of 4 or more, with fewer than 4 of 5 positive psychological items) or high risk of poor outcome (score of 4 or more, with 4 or 5 positive psychological items) and their subgroup guides the decisions about to treatment. The tool is presented on one side of A4 and is quick to complete and score. The targeted treatment for those at low risk of poor outcome mirrors that provided by GPs (above). For those at medium risk of poor outcome, the physiotherapist will carry out a comprehensive assessment (including a physical assessment) and negotiate an individualised treatment plan which specifically targets the patient's physical prognostic indicators using evidence-based treatments, including advice and explanation, reassurance, education, exercise and manual therapy. The number of treatment sessions is flexible, depending on the needs of the individual patient. Patients at high risk of poor outcome will receive a package of care that addresses psychological obstacles to recovery, in addition to addressing the physical symptoms. This consists of a comprehensive biopsychosocial assessment, including a physical examination and structured identification of individual obstacles to recovery, followed by evidence-based management similar to that described for patients at medium risk of poor outcome but taking a more focused cognitive-behavioural approach. Physiotherapists with additional skills drawn from cognitive-behavioural approaches, to identify and address psychological distress, and enhanced communication skills including motivational interviewing techniques and collaborative goal setting will treat these patients, within the context of an intervention promoting activation, return to normal activities (including work) and management of future recurrences. There is a specific focus on the prognostic indicators identified by the subgrouping tool and the number of treatment sessions is flexible, depending on the needs of the individual patient. Where indicated, patients in the medium and high risk subgroups will be referred onwards for consideration for investigations or secondary care interventions.

### Multi-component quality improvement intervention

We designed our improvement intervention with the view that this could, and should, be reflected on and amended in response to feedback. We reviewed previous studies of implementation research [42] and specifically studies aiming to change the behaviour of health care practitioners in managing LBP [11,26-31,43]. Broadly, these suggested that practitioner-orientated interventions (education, academic detailing, reminders, peer feedback, using educationally influential practitioners) tended to be more effective than those aimed at organisations or patients, that active educational interventions (that include patient examples, practice time, role play, simulation, feedback and review) that directly engage practitioners and multifaceted interventions (combining elements of both educational and organisational interventions) are more likely to be effective. Whilst no single theory of behaviour change was used solely to guide the intervention [44] we used the data from phase 1 on practitioners' attitudes, beliefs and confidence in managing LBP, their treatment orientations from the PABS measure, baseline reported practice (in response to standardised patient vignettes) and actual practice patterns to guide the content of the quality improvement intervention.

Features of the intervention with GP practices and GPs:

a) Two interactive, practice-based group educational sessions referred to as 'Best Practice Updates' for GPs, practice managers and practice nurses. Key messages include diagnostic triage and red flag assessment, the role of imaging, reassurance and advice, activity promotion, sickness certification, return to work and subgrouping for targeted treatment. The subgrouping tool will be introduced and the recommended targeted treatments discussed. Discussion on operational aspects of the study will be included, such as the way in which referral rates to physiotherapy will be monitored regularly so that adequate additional support can be provided, if needed.

b) Embedding the 6-item subgrouping tool in a pop-up computer screen, within the GP EMIS computer systems. The electronic tool is activated whenever a Read Code indicating a non-specific LBP problem is entered by the GP, reminding the GP about the study and prompting completion of the subgrouping tool. A treatment recommendation is automatically generated to support decision-making. We will also offer a paper-based version for those GPs who wish to use it.

c) Educationally influential practitioners (opinion leaders) suggested by GPs (a consultant rheumatologist, a GP with special interests (GPSI) in musculoskeletal problems from the local area) will lead the Best Practice Updates and the GPSI will take a lead role in regular communication with each practice.

d) Support for the initiative by key leads within the organisation (Primary Care Trust), including a lead GP from the Professionals Executive Committee (PEC) and the Chair of the PCT. Two of these individuals will also provide ongoing support for the study as members of the Steering Group.

e) Identification of a volunteer 'link' GP from each practice who will be the key person through which communication about the study will flow. Additional funding for one clinical session per month will support this role.

f) Providing each GP Practice with a study pack containing full details about the IMPaCT Back study.

g) Practice based feedback and individual feedback: Data from phase 1 from GP questionnaires and from actual practice data will be analysed and key findings summarised in the Best Practice Updates, comparing each practice with other participating practices (anonymised) and comparing each GP with other GPs within the same practice (not anonymised). This feedback will be provided in written form in the Best Practice Updates and used to stimulate peer discussion about variation in the management of LBP within each practice.

h) Regular reminders and audit/feedback: we will provide regular email and written communication about the study, and about patient recruitment and use of the subgrouping tool per GP and per practice. The timing of the reminders and regular feedback is anticipated to be monthly, but will be guided by feedback from practices. Reminder stickers about the study will be made available for those practitioners who wish to use them for their computer monitors.

i) Patient case-based discussion: depending on progress and on availability within the practices, we aim to facilitate further group-based practice meetings to focus on patient care, using a patient as an example to show the potential benefits of the sub-grouping for targeted treatment approach in practice. These will be led by a physiotherapist to whom the GP practice refers patients, in order to facilitate patient-based communication between the practice and the associated physiotherapy services.

j) Funding support: As well as the additional funding to support the GP link for the study in each practice (one clinical session per month), each practice will also be given £10 per patient identified, in acknowledgement of the small increase in time that it takes to determine eligibility of patients, to mention the study to the patient, and to complete the 6-item version of the sub-grouping tool.

k) Offers of additional one-to-one or small group educational meetings: each GP practice and GP will be offered further contact with a GP lead in the study team and the study co-ordinator, at regular intervals throughout phase 2 and 3 to review the use of sub-grouping tool, the aims of the study, the targeted treatments or any other study related issue.

Features of the intervention with physiotherapy services and physiotherapists:

a) Pre-training programme reflective activities/reading.

b) Stepped education and training programme consisting of a 3 day course for those delivering the targeted treatment for patients classified as at low and medium risk of poor outcome, including use of the 9-item subgrouping tool, identifying and managing patients with physical obstacles to recovery, best practice for LBP and nerve root pain. A further 6 days of training for those physiotherapists treating patients at high risk of poor outcome. These additional 6 days focus on identifying and managing psychological obstacles to recovery and development of skills through role play, case discussion, audio and video training materials and simulated patients. The training programme is supplemented with comprehensive manuals but is designed to be as experiential, skills-based and interactive as possible and includes discussion of operational aspects of the study. Funding support to cover backfill for time taken out from clinical practice for the purposes of training will be provided and training linked into Continuous Professional Development plans for individuals through negotiation with physiotherapy service managers.

c) Individual feedback and review in monthly mentoring sessions over 12 months following the training programme, the first six months led by the training leads and the second six months by senior physiotherapists and IMPaCT Back participants within the PCT. This was included as ongoing mentoring is likely to be the most effective way of consolidating and further developing knowledge, skills and confidence in the new subgrouping for targeted treatment system.

d) The subgrouping tool integrated within the physiotherapy assessment for patients with back pain and the targeted treatment pathways embedded into the ongoing care pathways for patients.

e) Educationally influential practitioners (opinion leaders) leading the training and mentoring programme, including a consultant clinical psychologist, consultant physiotherapist specialising in pain management, an extended scope physiotherapist in a spinal specialist role, a consultant rheumatologist, a GPSI and a disability advisor.

f) Support for the initiative by key leads within the organisation (Primary Care Trust), including the Chair of the PCT and physiotherapy service managers.

g) Study pack of all study information and documentation.

h) Study newsletter to provide feedback to the participating physiotherapists on the progress of the study.

i) Monthly monitoring of referral rates to physiotherapy and patient waiting times in order to identify and react promptly with funding support to increase physiotherapy response to any increase in demand for the service.

j) Agreed onward care pathways for those patients felt by physiotherapists to require the services of secondary care specialists.

### Phase 3: Observation of clinical practice and patient outcomes following implementation of the new care system

In phase 3, health care practitioners will be able to use the subgrouping tools and the targeted treatment approaches for LBP patients as introduced in phase 2. We will collect data on health care practitioners' back pain-related attitudes and behaviours, patients' clinical outcomes and health care resource use after the introduction of the new subgrouping for targeted treatment system.

#### a) Health care practitioners' attitudes and behaviour and care processes

We will repeat the questionnaire survey of participating GPs and physiotherapists in order to assess changes in their behaviour, attitudes and beliefs about back pain and its management after completion of the training (phase 2) and implementation of the new care systems (phase 3). We will record their reported behaviour in response to the same two clinical case vignettes of real patients with non-specific LBP, and their actual clinical behaviour, from GP medical record reviews and physiotherapy case report forms (in order to compare them with the same data from phase 1).

#### b) Clinical outcomes for patients

A new cohort comprising a consecutive sample of patients consulting with LBP at each participating practice will be recruited in phase 3 for a period of 12 months. The longer timescale in phase 3 is deliberately chosen so that we can study the pattern of use of the subgrouping for targeted treatment systems over time. As time progresses, the use of the new tools and targeted treatment may reduce, as practitioners may tend to go back to previous patterns of practice, and we aim to monitor this.

When the GP enters a relevant back pain Read Code into their clinical computer, the pop-up screen will include the same items as in phase 1 plus the brief (6 item) subgrouping tool, which, once completed, will yield a targeted treatment recommendation for the GP and patient to use to inform their decision-making. Similarly, if the patient is referred to physiotherapy, their assessment at the first visit with the physiotherapist will include use of the subgrouping tool (9 item version), which will yield a recommendation about targeted treatment.

Patient recruitment in phase 3 will be the same as the procedures described for phase 1. Participants who consent to further contact will receive a follow-up questionnaire at 2 months and 6 months (see figure 2). Those patients who reconsult in phase 3, who have previously been invited to take part in phase 1 will not be re-invited. The same patient outcome measures as phase 1 will be used in phase 3 (see Table 2).

#### c) Outcomes for the estimation of cost-effectiveness

The collection of cost and outcome data within phase 3 will mirror the approaches in phase 1, i.e. generic measurement of preference-based health-related quality of life using the EQ-5D and cost components that will allow for the estimation of back pain-specific and generic costs associated with the 6 month period following participants' initial consultation. In addition to the estimate of cost-effectiveness for the subgrouping approach (see 'Data analysis' section) we will explore whether there is evidence for sustainability. We anticipate that improved primary care for patients with LBP will reduce demands on secondary care for further investigation and management.

### Sample Size

#### Health care practitioners

We aim to involve a minimum of 20 GPs and 20 physiotherapists in the questionnaire surveys of phases 1 and 3, from the participating GP practices and related physiotherapy services.

#### Patients

Scoping work in preparation for this study has provided us with information on rates of consultation and current referral pathways, by which we can assess the expected recruitment and determine the power we will have to detect clinically important changes in patient outcome. The consultation rate for back pain in local general practices ranges from 6-10% annually. We aim to invite 7 GP practices (population of approx 45,000 adults aged 18 years or over) from which we expect at least 2,700 LBP consulters per year. Previous research suggests that approximately 50% of patients will be willing to consent to completing the questionnaires [45]. Based on these figures, we anticipate that within the timeline of the study (across both phases 1 and 3), recruitment of 1000 patients should be feasible.

A difference of 2.5 points in RMDQ [37] change scores is considered to be a minimum clinically important difference [46]. We wish to test the superiority of the subgrouping for targeted treatment system (phase 3) over usual primary care (phase 1) in relation to this 2.5 point difference for those patients in the high and medium risk subgroups, and to test for non-inferiority of the new care system compared to usual care in the low risk subgroup. A weighted sample size (based on the timeline ratio of 1:2 (phase 1: phase 3) of 50 patients (phase 1) and 100 patients (phase 3) is powered at about 80% to detect a 2.5 point difference, assuming a standard deviation of 5 and using a 5% two-tailed significance level. Based on our pilot work, we expect 20% of back pain patients to be classified as at "high-risk" of poor outcome, 40% to be at "medium-risk" and 40% at "low-risk". Thus, our expected study size of 1000 patients will enable us to evaluate this clinical difference across the three risk subgroups, and has 80% power to detect a 'small' effect size of about 0.2, equivalent to a mean difference in RMDQ scores of approximately 1, over the total study population (taking into account 20% loss to follow up).

### Data analysis

#### Health care practitioners' attitudes and behaviour and care processes

A descriptive summary of the characteristics of the participating GPs and physiotherapists will be provided. To assess differences in health care practitioners' attitudes between phase 1 and phase 3, we will compare baseline mean scores and follow-up mean scores for the self-confidence and PABs scales using matched-analyses techniques (e.g. paired t-test) based on before (phase 1) and after (phase 3) matching of scores to individual health care practitioners. The matched-analyses are based on evaluating the same health care practitioners before and after; a sensitivity analysis using unmatched approaches (e.g. unpaired t-test) will be used if the pool of practitioners in phase 3 is not exactly the same as that in phase 1. As well as providing the best estimate of the true mean difference, we will also present 95% confidence interval estimates for the difference and give p-values for the statistical tests performed. Mean changes in attitudes scores (3 questionnaires: before the quality improvement interventions, immediately after the quality improvement interventions and 12 months after the quality improvement interventions) will also be described.

Behaviour measures, relating to the two case vignettes, follow categorical or textual response formats and differences over time will be described. Health care practitioner behaviour differences between time points will also be statistically evaluated by comparing data of their actual clinical practice, e.g. health care consultations, medication prescriptions, sickness certification and patterns of referral obtained from medical record downloads for patients who consent to record reviews and from physiotherapists' case report forms. Furthermore, GP responses to the computer "pop-up" prompt on 'chronicity risk' (low; medium; high) and presence of 'yellow flags' (no; yes) will be compared to patient questionnaire responses to the subgrouping tool; the percentage agreements would be expected to be higher after the quality improvement intervention. These analyses, based on patient measures, are unmatched and will follow between-group techniques i.e. unpaired t-test, chi square test and regression methods (as described below for clinical outcomes).

#### Clinical outcomes for patients

The primary outcome measures of patient benefit will be back pain-related disability (measured on the RMDQ [37] and pain catastrophising (measured on the catastrophising subscale of the Coping Strategies questionnaire [38] at 6 month follow up. Primary analysis of the patient data will follow the principles of "intention to treat", to compare outcomes of patients in phase 1 (pre-intervention) to those of patients in phase 3 (post-intervention). A "per-protocol" analysis, based on the evaluation of phase 3 patients who were treated with strict adherence to the subgrouping tool allocation rule (versus all phase 1 patients) will be carried out as a secondary comparison. Estimates of treatment effects, i.e. comparative outcomes for phase 1 (pre-intervention) versus phase 3 (post-intervention), with 95% confidence intervals will be calculated using linear regression (for numerical outcomes) and logistic regression (for categorical outcomes). Analyses will be carried out both unadjusted and adjusted for baseline imbalances in certain variables i.e. age, gender and baseline values of key outcomes. Statistical adjustment is necessary as this is an observational, before and after study design, and thus open to the possibility of confounding bias; adjustment will ensure parity of key baseline patient characteristics across phases 1 and 3. Analyses will be carried out at each patient follow up time point (2 and 6 months) for primary and secondary outcome measures; the primary endpoint is 6 months. Analyses will be performed on available data, and on imputed datasets based on the method of multiple imputation [47]. We will use STATA and SPSS for the analyses.

#### Determining the cost-effectiveness of subgrouping for targeted treatment

Consideration of cost-effectiveness for any intervention or policy change requires an incremental approach, where the focus of analysis is on the joint estimation of the *differences *in costs and benefits between the new process (in this case, phase 3 following the implementation of subgrouping for targeted treatment systems) and the standard process (phase 1).

The primary health economic analysis will focus on the estimation of incremental cost-utility from an NHS perspective, in line with current national guidelines [39]. Accordingly, the appropriate cost components for the base case analysis will consist of all items of back pain-related resource use within the NHS. Quality-adjusted life years (QALYs) will be calculated by applying area-under-the-curve techniques to EQ-5D scores at baseline, 2 months and 6 months [48], assuming linear interpolation between consecutive data collection points. Differences (phase 3 minus phase 1) in costs and QALYs will be expressed using the incremental cost-per-QALY ratio, which provides an estimate of the additional cost required to gain each additional unit of outcome (1 QALY is interpreted as 1 year spent in full health). The incremental ratio is merely a point estimate and, therefore, it is necessary to address uncertainty. In line with current recommendations, uncertainty will be addressed using bootstrapping techniques, cost-effectiveness planes and acceptability curves [49,50]. As with the clinical outcomes for patients multiple imputation techniques will be used to address the issue of incomplete data [47].

To explore the robustness of our findings, a number of sensitivity analyses will be performed. Data collected as part of this study will enable other perspectives to be considered with regard to the estimation of cost-effectiveness. Moving beyond health care resources attributable to the NHS, a broader 'societal' perspective will incorporate the indirect costs of reduced productivity at work due to low back pain and also costs incurred within the private health care sector. A second sensitivity analysis relating to costs will incorporate generic resource use, rather than just those relating to back pain. With regard to outcome valuation, an alternative UK-derived preference-based measure of health status, the SF-6D, will be considered [51]. Finally, a complete-case analysis will be performed to assess the influence of missing data and the value of adopting multiple imputation methods.

### Other analyses

Qualitative research will be nested within the IMPaCT Back study. Data collection will be via semi-structured interviews with GPs, physiotherapists and managers. Qualitative thematic analysis will be aided by the NVivo data management system and highlight factors that promote or inhibit the implementation of the new subgrouping for targeted treatment system. We are adopting the Normalisation Process Theory (NPT) as the conceptual framework to assess the trajectory of the new subgrouping for targeted treatment system from its introduction to early implementation and longer-term maintenance. The NPT [52] focuses on how new practices are embedded and integrated into their social contexts and it allows a systematic and in-depth understanding of the organisational and social processes that help complex interventions become embedded.

## Discussion

Although it is generally accepted that randomised controlled trials provide the highest level of evidence in healthcare research, we opted for a pre-post design as it was more appropriate to study real-life implementation of a complex intervention, using multiple components and targeting multiple outcomes. Using a quasi-experimental design, the IMPaCT Back study will introduce, support and evaluate a new care system for assessing and managing LBP patients in primary care; subgrouping for targeted treatment. As such, the overall evaluation of this quality improvement study will take account of a wider range of factors than is usually seen within conventional randomised controlled trials. This includes health care practitioner attitudes and behaviour (both reported behaviour and actual behaviour), evaluation of clinical care processes, patients' clinical outcomes and cost-effectiveness of the new system.

The fluid nature of systems and structures within the NHS means that health care provision is constantly changing, and this introduces an added layer of complexity into our analysis of change in this study. Therefore, given the before/after design of the study, it may not be possible to definitively conclude that any changes we see in phase 3 are a direct result of the interventions from the IMPaCT Back study. We will, therefore, monitor other changes at national level (such as the production of new guidelines) and at local level, such as the commencement of new services for LBP patients within the PCT and changes in referral rates and waiting lists for clinical care.

There are limitations to the pre-post study design, including that any changes seen in phase 3 may be due to other influences or due to change in GP and physiotherapist behaviour during the study, for reasons other than the IMPaCT Back study. Potential Hawthorne effects are likely to be minimised given the long observational period (6 months in phase 1 and 12 months in phase 3).

This study directly addresses one of the key recommendations from the NICE [15] LBP guidelines: to evaluate screening protocols and test their effectiveness in targeting treatments for patients. If the data favour phase 3 of the IMPaCT Back study, this will provide valuable information about brief and feasible ways to screen patients with LBP in primary care, and improve their outcome by using information about their risk status at consultation.

The results of this study will be made available for use by clinicians, service managers, and commissioners of care services.

## Competing interests

The authors declare that they have no competing interests.

## Authors' contributions

NF, EH conceptualised and designed the study and secured funding. RM, NF, ML, DW wrote the full protocol. RM and NF wrote the first draft of this manuscript. All authors contributed to revisions of this manuscript, have read and approved the final manuscript and take public responsibility for its content. NF is the principal investigator, RM is the study co-ordinator, ML the biostatistician, DW the health economist, JY the research nurse, CD the clinical liaison physiotherapist and EH the chief investigator.

## Pre-publication history

The pre-publication history for this paper can be accessed here:

http://www.biomedcentral.com/1471-2474/11/186/prepub
